# Trainee resident participation in health research in a resource-constrained setting in south-eastern Nigeria: perspectives, issues and challenges. A cross-sectional survey of three residency training centres

**DOI:** 10.1186/1472-6920-12-40

**Published:** 2012-06-13

**Authors:** Boniface Ikenna Eze, Cajetan Uwaturuonye Nwadinigwe, Justin Achor, Emmanuel Nwabueze Aguwa, Anthony Mbah, Francis Ozoemena

**Affiliations:** 1 Department of Ophthalmology, University of Nigeria Teaching Hospital, Ituku-Ozalla, Enugu, Nigeria; 2 National Orthopaedic Hospital, Enugu, Nigeria; 3 Federal Neuro-psychiatric Hospital, Enugu, Nigeria; 4 Department of Community Medicine & Public Health, University of Nigeria Teaching Hospital, Ituku-Ozalla, Enugu, Nigeria; 5 Department of Pharmacology and Therapeutics, University of Nigeria Teaching Hospital, Ituku-Ozalla, Enugu, Nigeria; 6 Department of Anatomy, University of Nigeria Teaching Hospital, Ituku-Ozalla, Enugu, Nigeria; 7 Department of Ophthalmology, University of Nigeria Teaching Hospital Ituku-Ozalla, PMB 01139, Enugu, Nigeria

**Keywords:** Trainee residents, Health research, Participation, Attitudes, Barriers, Nigeria

## Abstract

**Background:**

The participation of trainers and trainees in health research is critical to advance medical science. Overcoming barriers and enhancing incentives are essential to sustain a research culture and extend the frontiers of medical education. In this study, we investigated the roles of individual and system factors influencing trainee resident participation in health research in Enugu, south-eastern Nigeria.

**Methods:**

This cross-sectional survey of trainee residents was conducted across three residency training centres in Enugu, Nigeria, between February and March, 2010. The number and speciality distribution of trainee residents were determined from personnel records at each centre. A 19-item questionnaire was used to record demographic characteristics, research training/experience, and attitudes toward and perceived barriers to health research. Data were analysed to yield frequencies, percentages and proportions. Values of *p* < 0.05 were considered significant.

**Results:**

The response rate was 93.2%. The respondents (n = 136) comprised 109 males and 27 females. Their mean ± standard deviation age was 35.8 ± 5.6 years (range: 25–53 years). Participation in research was significantly associated with previous research training [odds ratio (OR): 2.90; 95% confidence interval (CI): 1.35–6.25, *p* = 0.003, β = 22.57], previous research participation (OR: 2.21; 95% CI: 0.94–5.29, *p* = 0.047, β = 22.53) and research publication (OR: 2.63; 95% CI: 1.00–7.06, *p* = 0.03, β = 22.57). Attitude towards research was significantly influenced by perceived usefulness of research in patient care (OR: 7.10; 95% CI: 3.33–15.13, *p* = 0.001), job promotion (OR: 8.97; 95% CI: 4.12–19.53, *p* = 0.001) and better understanding of disease (OR: 21.37; 95% CI: 8.71–54.44, *p* = 0.001). Time constraints (OR: 0.06; 95% CI = 0.025–0.14, *p* = 0.001), funding (OR: 0.028; 95% CI: 0.008–0.10, *p* = 0.001) and mentorship (OR: 0.086; 95% CI: 0.36–0.21, *p* = 0.001) were significant barriers to research participation.

**Conclusions:**

System and individual factors are significant incentives to research participation, while system-derived factors are significant barriers. Pre-residency research, dedicated research time, adequate research funding and commensurate research mentorship rewards are instructive. Prospective longitudinal studies are warranted to confirm these findings.

## Background

Health research, including basic science and clinical research, is fundamental to establish the scientific foundations of clinical care and to translate basic research findings into tangible benefits for the healthcare system [1,2]. Sustained generation and unhindered dissemination of the findings of quality health research, in addition to advancing the knowledge of disease processes [1], are critical components of evidence-based medicine [1-3], for informed changes to public health policy [1,4] and to enhance researchers’ critical appraisal skills [1,4].

Despite the enormous benefits derived from health research [4], there is a health system-wide discrepancy between the realisation of the need for health research and the realities of its implementation [3,5]. This gap has been attributed to several factors, notably an insufficient number of adequately trained researchers (e.g., academic physicians, clinical researchers, physician-scientists, clinical investigators and physician-investigators) [6,7]. Previous studies have also suggested obstacles at the individual level, including gender [1,5], time constraints [1,5,6], a lack of interest [3,5], poor awareness [6,8] and inappropriate remuneration [1,7]. System-related disincentives, including poor research funding [1,7], inadequate research mentorship [9-11], insufficient statistical support [1,3,6], poor research training [2,8], restricted access to literature [1,6], a lack of autonomy [12] and bureaucracy/politics [12], also constitute major barriers to the participation in research and a future career in academic medicine. Specifically, males more frequently participate in research and, by extension, have greater prospects for future careers in academic medicine [5]. In Nigeria, there is a pro-male bias in enrolment into residency training programmes [13-15], and this has favourable implications for residents in terms of participation in research [1,5] and choice of post-residency research careers [6]. Non-utilisation of research findings by healthcare providers and health policy makers, either because of impeded access [1,4] or limited understanding of their clinical or health policy implications [1,4], has a negative impact on future research effort and output.

Globally, there exists a marked North–South divide, favouring the North, in terms of the awareness, output and implementation of health research [4]. Consequently, particularly in developing African countries where the output of health research is comparatively low [16,17], modifications of clinical practice and health policy are often inappropriately based on imported research results. Therefore, to resolve this situation, there is an urgent need for research that identifies the indigenous determinants of participation in health research that affect all levels of health research, not just physician researchers. To partly fulfil this need, we conducted an exploratory cross-section pilot survey in which we sought to identify which factors experienced before/during residency at the individual and system levels influenced trainee resident participation in health research across three tertiary training centres in Enugu, south-eastern Nigeria. Our results will assist undergraduate and postgraduate medical educators in Enugu, as well as those in similar settings elsewhere in Nigeria and other countries. The findings should also stimulate similar research in Nigeria, and other developing countries, to replicate at least some of our findings, and also identify other factors that may be specific to other academic institutes in other countries.

## Methods

### Background

Nigeria’s south-east geo-political zone, predominantly populated by ethnic Ibos, is one of six geo-political zones in Nigeria. The zone comprises five states of Abia, Anambra, Ebonyi, Enugu and Imo. The inhabitants of the zone are mainly traders, civil servants and artisans. Enugu state, established in 1991, is one of 36 states of the Federal Republic of Nigeria. Enugu City, the administrative capital of Enugu state, contains numerous academic institutions devoted to medical and non-medical fields. The duration of undergraduate medical training in Nigeria is 6 years. To qualify for registration as a general medical practitioner, a 1-year medical internship after graduation is mandatory. Residency training lasts for 4–6 years. During training, the residents sequentially undertake the Primary, Part I, and Part II (Final) Fellowship examinations in their chosen specialty. While the individual universities certify their medical graduates, the Nigerian Medical and Dental Council accredit and regulate undergraduate medical training. The National Postgraduate Medical College, part of Nigeria’s Federal Ministry of Health, and the West African Postgraduate Medical College, an affiliate of the West African Health Community, are the two agencies responsible for training and certification of residents, and for accreditation/regulation of residency training programmes.

All of the fully accredited training centres that offer residency training in some or all of the 14 locally available residency training programmes are government-owned except for one privately owned centre—The Eye Foundation Hospital, Lagos—which is accredited for residency training in ophthalmology. Three of these centres, The University of Nigeria Teaching Hospital (UNTH), The National Orthopaedic Hospital Enugu (NOHE) and The Federal Neuro-psychiatric Hospital (FNPH), are located in Enugu. The UNTH offers training in all 14 specialties; the NOHE provides training in orthopaedic, trauma and plastic surgeries, while the FNPH only offers training in psychiatry.

In Nigeria, in addition to a mandatory research component of postgraduate training, trainee residents participate in various types of research as part of their extra-curricular academic activities. These research activities are frequently hospital-based epidemiological surveys and observational studies, and infrequently interventional studies and clinical trials. However, a few trainee residents participate in population-based surveys.

### Ethics

Before starting this study, we obtained ethical clearance from the Ethical Committees/Institutional Review Boards at all three study centres. Before recruitment, oral informed consent was obtained from potential participants, after assuring them of their anonymity and the confidentiality of the study results.

### Study design

Between February 1 and March 31, 2010, we conducted a cross-sectional questionnaire-based survey of trainee residents at three residency training centres (UNTH, NOHE, and FNPH) in Enugu, south-eastern Nigeria. Background information on the number and specialty distribution of the trainee residents was obtained from the human resources departments of the three study centres. The chief (most senior) resident in each department/specialty was identified and approached to assist in the logistics of study administration.

### Questionnaire and questionnaire development

The study instrument was a 19-point pretested, open-ended, self-administered questionnaire. The questionnaire was adapted from instruments used in similar surveys conducted in Pakistan [1] and Japan [2] after searches of PubMed failed to identify similar surveys in Nigeria or similar countries. The questionnaire was adapted to ensure it was relevant for the study environment, with consensus from all participating investigators. The questionnaire comprised five sections, focusing on the participants’ socio-demographic characteristics, previous research training/experience/publication, attitudes/perceptions to health research, perceived barriers to participation in health research, and future research career plans.

The questions on socio-demographic characteristics, research training/publication/experience, and future research career plans were answered using dichotomous (Yes/No) responses. The questions on attitude/perceptions and personal barriers were answered using a 5-point Likert-like scale where 1 = I strongly agree, 2 = I agree, 3 = I neither agree nor disagree, 4 = I disagree and 5 = I strongly disagree. Before analysis, the responses were collapsed into three categories, where ‘I strongly agree’ and ‘I agree’ were taken as positive (i.e., Yes), ‘I strongly disagree’ and ‘I disagree’ were taken as negative (i.e., No) and ‘I neither agree nor disagree’ was taken as a neutral response.

To confirm construct validity and psychometric reliability, and to ensure the questionnaire was appropriate for the study objectives, the questionnaire was pretested on a cohort of trainee residents not affiliated to the study centres. Further qualitative approaches to ensure reliability and validity included self-administration, which guaranteed anonymity and confidentiality, and avoidance of leading. Explicit operational definitions of the study variables were provided to enhance the validity of the survey instrument. Structural modifications identified in the pre-test were also implemented, where necessary, before finalizing the questionnaire.

### Exclusion criteria

Trainee residents in the fields of Community Medicine (Public Health) and Basic Health Sciences (Anatomy, Physiology and Medical Biochemistry) were excluded from this study because the research-focused nature of their training confers a disproportionate advantage for these residents over other residents [1]. Residents who voluntarily declined to participate were also excluded.

### Definition of variables

Participation in health research was defined as a significant direct involvement in collection, analysis and interpretation of research data, and a significant contribution to the intellectual content of the resulting manuscript. Participation in data collection alone was not considered as participation in health research. Research training was defined as formal training in research methodology during the undergraduate or postgraduate period. Research presentation was defined as presentation of research results in a regional, national or international conference. Research publication was defined as publication of research results in a peer-reviewed regional, national or international journal.

### Data analysis

Data were entered and analysed using Statistical Package for Social Sciences (SPSS) software version 15.0 (SSPS Inc., Chicago, IL, USA). The accuracy of data entry was confirmed by selectively re-entering data from 30 randomly selected questionnaires. Descriptive analyses were performed to yield frequencies, percentages and proportions. The participants were then categorised into two groups, as those currently participating in research, and those not currently participating in research. Statistical tests were used to identify significant differences between these two groups and identify factors associated with participation in research. The influence of current participation in research on future (post-residency) research career choice was also explored. The *χ*^2^ test was used for categorical variables and *t* test for non-categorical variables. In all cases, odds ratios (OR) with 95% confidence intervals (CI) associated with *p*-values < 0.05 were considered statistically significant. Characteristics that showed significant crude associations with participation in health research in univariate analyses were incorporated into a multiple linear regression model to identify factors showing independent associations with participation in health research.

## Results

Of 151 trainee resident across the three study centres, 5 (3 males and 2 females), all from UNTH, declined to participate. Of 146 trainee residents who consented to participate, 136 returned completed questionnaires [response rate, 93.2% (136/146)]. Of the respondents, 90 (66.2%) were affiliated to UNTH, 30 (22.1%) to NOHE and 16 (11.8%) to FNPH. Since the survey was anonymous, the gender distribution of the 10 non-respondents could not be determined.

### Socio-demographic profile

The participants comprised 109 (80.1%) males and 27 (19.1%) females (sex ratio = 4:1). Their mean ± standard deviation age was 35.8 ± 5.6 years (range: 25–53 years). The observed gender difference was statistically significant (80.1% *vs*. 19.9%, *p* < 0.05). The age and sex distribution of the study participants is shown in Table 1. The respondents consisted of significantly more junior than senior residents (68.4% *vs*. 31.6%, *p* < 0.05), with larger proportion in surgery and allied specialties (86, 63.2%) than in general medicine and allied disciplines (50, 36.8%).

**Table 1:** Age and sex distribution of 136 trainee residents

**Age (years)**	**Sex**		**Total**	%
	M	F		(n = 136)
20–25	1	0	1	0.7
26–30	11	10	21	15.4
31–35	36	9	45	33.1
36–40	26	4	30	22.1
41–45	30	2	32	23.5
46–50	1	1	2	1.5
51–55	4	1	5	3.7
Total (%)	109 (80.1)	27 (19.9)	136	100

### Previous research training/participation/publication/experience

The proportions of trainee residents currently participating or not participating in research were as follows: UNTH, 43 (68.3%) vs 47(64.4%); NOHE, 11 (17.5% vs 19 (26.0%); and FNPH, 9 (14.3%) vs 7 (9.6%). Of the respondents, 91 (66.9%) had received undergraduate research training, 57 (41.9%) had received post-graduate research training, and 38 (27.9%) had received both. Overall, 99 (72.8%) of the respondents had participated in health research during undergraduate or postgraduate education while 37 (27.2%) had not. Sixty-three (46.3%) respondents were currently participating in research while 73 (53.7%) were not. Of 37 (27.2%) respondents who had previously submitted an original research article for publication, 26 (19.1%) had their submitted articles published. Thirty five (25.7%) respondents reported that they had previously presented their research. Seven (5.1%) respondents read scientific journals/periodicals daily, 29 (21.3%) weekly, 10 (7.4%) every 2 weeks, 23 (16.9%) monthly and 67 (49.3%) read journals less than once every month.

Table 2 reports the factors associated with current participation in research. In univariate analyses, the trainee residents who were currently participating in health research (n = 63) were significantly more likely than non-participants (n = 73) to have received previous research training (OR: 2.90, 95% CI: 35–6.25, *p* < 0.05), participated in previous research (OR: 2.21, 95% CI: 0.94–5.29, *p* < 0.05), published a research article (OR: 2.63, 95% CI: 1.0–7.06, *p* <0.05) or be junior residents (OR: 0.20, 95% CI: 0.08–0.48, *p* < 0.05).

**Table 2:** Factors associated with current participation in health research

**Characteristics**	**Total**	**Percent currently**	**Percent not currently**	***Odds ratio*(95% CI)**	***P-value***
	(n = 136)	participating	participating		
	n(%)	in research	in research		
		(n =63)	(n = 73)		
Sex					
-Male	109(80.1)	50(79.4)	59(80.8)	0.91(0.36–2.30)	0.832
-Female	27(19.9)	13(20.6)	14(19.2)		
Age (years)					
- < 35	56(42.6)	24(38.1)	34(46.6)	0.71(0.34–1.48)	0.319
- ≥ 35	78(57.4)	39(61.9)	39(53.4)		
Specialty					
-Surgery and allied	86(63.2)	39(61.9)	49(67.1)	1.06(0.48–1.33)	0.857
-Medicine and allied	50(36.8)	24(38.1)	24(32.9)		
Cadre					
-Junior resident	93(68.4)	32(50.8)	61(83.4)	0.20(0.08–0.48)	<0.05*
- Senior resident	43(31.6)	31(49.2)	12(16.4)		
Any previous undergraduate	91(66.9)	39(61.9)	52(71.2)	0.66(0.30–1.43)	0.249
research training? Yes/No	45(33.1)	24(38.1)	21(28.8)		
Any previous postgraduate	57(41.9)	35(55.6)	22(30.1)	2.90(1.35–6.25)	0.003*
research training? Yes/No	79(58.1)	28(44.4)	51(69.9)		
Any previous participation	99(72.8)	51(80.6)	48(65.6)	2.21(0.94–5.29)	0.047*
in research? Yes/No	37(27.2)	12(19.0)	25(34.2)		
Any previous research publication?	26(19.1)	17(27.0)	9(12.3)	2.63(1.0–7.06)	0.03*
Yes/No	110(80.9)	46(73.0)	64(87.7)		
Any previous research presentation?	35(25.7)	21(33.3)	14(19.2)	1.92(0.83–4.51)	0.097
Yes/No	101(74.3)	46(73.0)	59(80.8)		
Any previous writing of	95(69.9)	48(76.2)	47(64.4)	1.77(0.78–4.03)	0.135
research protocol? Yes.	41(30.1)	15(23.8)	26(35.6)		
How often do you read					
health journals?					
- at least once every 2 weeks	46(26.5)	21(33.3)	15(20.5)	1.93(0.84–4.50)	0.092
- less than once every 2 weeks	90(73.5)	42(66.7)	58(79.5)		

*Significant

All four predictors of participation in health research identified in univariate analyses were retained as significant factors in multivariate regression, with regression coefficients (β) of 22.57 for previous research training, 22.53 for previous participation in research, 21.11 for previous research publication and −44.84 for junior residents. The negative regression coefficient for junior residents indicates that they were less likely than their senior colleagues to participate in health research. Senior residents with previous research training, who participated in previous research and who had previous research publication were more likely to be currently involved in health research.

### Attitudes to and perceptions of health research

We first examined whether the respondents were aware of the Ethical Principles for Medical Research Involving Human Subjects derived from the 1964 World Medical Association Declaration of Helsinki (Helsinki Declaration). Overall, 9 (6.6%), 50 (36.8%) and 77 (56.6%) respondents had satisfactory, fair or little awareness of the Helsinki Declaration. There was no significant difference in the proportion of respondents with at least fair knowledge (n = 59) of the Helsinki Declaration between participants and non-participants in research (31/63 *vs*. 28/73; OR: 1.107, 95% CI: 0.59–2.07, *p* = 0.7498).

The factors associated with attitudes to and barriers to participation in health research are shown in Table 3. Compared with non-participants, significantly more current participants in health research perceived health research as being useful in improving patient care (OR: 7.10, 95% CI: 3.33–15.13, *p* = 0.001), job promotion (OR: 8.97, 95% CI: 4.12–19.53, *p* = 0.001) and understanding of disease (OR: 21.37, 95% CI: 8.71–54.44, *p* = 0.001). Compared with current participants in health research, non-participants more frequently identified lack of dedicated research time (OR: 0.06, 95% CI: 0.025–0.14, *p* = 0.001), research funding (OR: 0.028. 95% CI: 0.008–0.10, *p* = 0.001) and mentorship (OR: 0.09, 95% CI: 0.36–0.21, *p* = 0.001) as barriers to participation in health research.

**Table 3:** Factors associated with trainee residents’ attitudes and barriers towards health research

**Statement**	**Percent currently**	**Percent not currently**	**Odds ratio (95% CI)**	***P-*value**
	**participating in research**	**participating in research**		
	**(n = 63)**	**(n = 73)**		
**Attitudes towards research**				
Promotes researcher’s critical	54(85.7)	64(87.7)	0.84(0.54–1.31)	0.8438
appraisal skills (Yes/Neutral/No)	4(6.3)	5(6.8)		
	5(7.9)	4(5.4)		
Improves patient care	47(71.4)	19(26.0)	7.10(3.33–15.13)	0.001*
	10(15.8)	24(32.9)		
	6(9.5)	30(41.1)		
Helps in job promotion	47(74.6)	18(24.7)	8.97(4.12–19.53)	0.001*
	6(9.5)	20(27.4)		
	10(15.9)	35(63.6)		
Helps to change health policy	51(81.0)	56(76.7)	0.91(0.57–1.45)	0.6931
	0(0.0)	7(11.1)		
	12(19.4)	10(15.9)		
Provides better understanding	54(85.7)	16(21.9)	21.37(8.71–54.44)	0.001*
of disease	5(7.9)	17(23.3)		
	4(6.3)	40(54.8)		
Provides financial benefits	28(44.4)	35(47.9)	0.8(0.44–1.47)	0.4712
	13(20.6)	18(24.7)		
	22(34.9)	20(27.4)		
through grants and loans				
Enhances researcher’s societal	31(49.2)	48(65.8)	0.65(0.37–1.12)	0.1174
standing	15(23.8)	12(16.4)		
	17(27.0)	13(17.8)		
**Barriers towards research**				
Lack of research awareness	49(77.8)	59(80.8)	0.83(0.52–1.32)	0.4318
	7(11.1)	5(6.8)		
	7(11.1)	9(12.3)		
Research is difficult	59(77.8)	65(80.8)	0.83(0.59–1.40)	0.6599
	1(1.6)	3(4.1)		
	3(4.8)	5(6.8)		
Lack of protected research time	17(27.0)	63(86.3)	0.06(0.025–0.14)	0.001*
	19(30.2)	0(0.0)		
	27(42.9)	10(13.7)		
Lack of research training	56(88.9)	67(91.8)	0.86(0.54–1.29)	0.4178
	2(3.2)	2(2.7)		
	5(7.9)	4(5.4)		
Lack of research funding	25(39.7)	70(95.8)	0.028(0.008–0.10)	0.001*
	10(15.9)	1(1.4)		
	28(44.4)	2(2.8)		
Lack of statistical support	59(93.7)	63(86.3)	0.94(0.61–1.45)	0.7675
	0(0.0)	2(2.7)		
	4(6.3)	8(11.0)		
Lack of research mentorship	26(41.3)	65(89.0)	0.086(0.36–0.21)	0.001*
	7(11.1)	3(4.1)		
	20(31.7)	5(6.8)		
Frustrations from rejection	38(60.3)	48(65.8)	0.79(0.47–1.33)	0.3783
Of submitted articles	11(17.5)	7(9.6)		
	14(22.2)	18(24.7)		

*Significant

### Post-residency research career intentions

Of the respondents, 87 (64.0%) intended to take up a research-related job after residency training, 7 (5.1%) opted for a non-academic appointment and 42 (30.9%) were undecided. Of the 87 respondents considering future research careers, 45 were currently participating in research and 42 were not. Current participation in research was not positively associated with post-residency career intentions (45/63 *vs*. 42/73; OR: 1.07, 95% CI: 0.64–1.79, *p* = 0.7928).

## Discussion

### Demographic characteristics

The respondents’ demographic characteristics showed a preponderance of males over females. The observed pro-male gender disparity, which is unlikely to be influenced by the gender profile of non-responders because of the high response rate, is consistent with the gender ratio of trainee residents in Nigeria [13-15] and Pakistan [1], and is consistent with the general trend in academic medicine [18]. However, a female gender dominance was observed in Boston, Massachusetts. [6]. The consistency between the current study with other Nigerian studies [13-15] suggests that the observed gender distribution is not due to a peculiar gender distribution of the respondents. The similarities in study setting and respondent characteristics between the present survey and the Pakistani study, and the differences to the Boston study may explain some of the observations. Furthermore, as previously reported [12,19-21], the female-specific conflict between academic medical career aspirations and domestic roles may also contribute to the gender disparity. The findings have notable implications for residency training programmes in Nigeria and in similar settings elsewhere that a customised, female-friendly, residency training structure may be needed to increase the number of females entering academic medicine.

### Factors associated with participation in heath research

The present report found that senior residents, post-graduate research training, previous participation in research and previous research publication are positively associated with current participation in health research among trainee residents.

Similar to the reports by Ulrich et al. in the United States [6] and Gill et al. in Canada [5], although different to other studies in Canada [12] and the United States [22,23], we found a direct relationship between the level of residency training and current participation in health research. While between-survey similarities/differences in study settings, design and respondent characteristics might explain the observed differences and similarities, the trend observed in the present survey may be due to the involvement of senior residents in mandatory dissertation-related research. To reverse this bias, the authors think that the research content of residency training should be increased coupled with mandatory research projects throughout the residency period.

The trends observed for previous research training, research participation and publication in this study reflect those reported in earlier studies [1,2,5,8]. These results emphasise the need to extend the frontiers of residency training, beyond acquisition of clinical knowledge and skills, to include regular research training and enforcement of graded participation in research commensurate with the resident’s status. To achieve these goals through the provision of research infrastructure and environment, we suggest the adoption of a national health research system framework that incorporates stakeholders across all levels of health research and implementation [24-26].

### Attitudes to health research

Trainee residents who were currently participating in research were significantly more likely to perceive health research as being useful for patient care, job promotion and better understanding of disease than those not currently involved in research. While these findings agree with the results of a similar survey in Pakistan [1] they differ from the conclusions drawn from a survey of practising physicians in Kyoto, Japan [3]. This could be explained by the similar characteristics of the respondents in our survey and those in the Pakistani survey and the marked differences from those in the Japanese survey. While the present study and the Pakistani study included trainee residents, the Japanese study focused on practising physicians. The findings have positive implications on evidence-based medicine and career progression, and suggest the need to further stimulate research interest by exposing residents to other potential uses of health research, outside patient care and career progression. In Nigeria, research and clinical practice, although co-existing, are almost parallel because of perceived barriers to research implementation. Therefore, to maximise the impact of health research on patient care, we must identify and overcome barriers to health research, and continuously equip clinicians with key practice and policy implications derived from synthetic research [25,26].

### Barriers to participation in health research

Respondents not currently participating in health research were significantly more likely to identify lack of dedicated time, poor research funding and the limited availability of research mentors as barriers to participation in health research. The lack of dedicated research time was acknowledged in other comparative surveys [1,5,6,21] as a major barrier to involvement in health research. However, Aslam et al. [8], in a descriptive report of research attitudes and practices of trainee residents in Pakistan, did not identify time constraints as a barrier to participation in research. The similarities in study design between the present study and the earlier surveys [1,5,6,21] and the differences from the study by Aslam et al. [8] probably explain these observations. To ensure residents have sufficient time dedicated to research, we propose that the residents are given at least 1 day every week devoted to research throughout the training period. To ensure this time can be effectively used for research purposes and to avoid conflict with other time-sensitive extra-curricular commitments, we suggest that the residents are allowed to choose this day.

Consistent with other surveys conducted in resource-constrained settings in low and medium income countries [1,4], and in developed economies [3,7], poor research funding has been identified as a fundamental barrier to participation in health research. Faced with competing public health needs, at times life-threatening, governments in developing countries often perceive research funding as expenditure rather than an investment in health. This policy often has tragic consequences for research-informed clinical practice, health policy modifications, as well as disease surveillance and control. Identification and adequate funding of clinical research funding priorities, coupled with positive re-orientation of governmental attitudes to research funding, are urgently needed to establish a critical mass of health care researchers necessary to sustain the health research enterprise. This is achievable through active multi-sector collaborations, within the context of a national health research framework, between all stakeholders involved in the production, dissemination and utilisation of health research.

The limiting availability of mentorship identified in the present study is consistent with that reported elsewhere [1,3,5-7]. This emphasises the need to motivate mentors by creating financial incentives and career progression opportunities for mentorship [7]. Additional measures to improve the effectiveness of mentoring may include the replacement of personal mentoring with peer group-based (collaborative) mentoring [9], and the provision of dedicated mentoring time for the mentors [7] and mentees [11].

In contrast with the finding by Ulrich et al. [6], the present survey did not find a positive association between participation in research and future research career intentions among trainee residents. This suggests that the participation in research is probably not due to any intrinsic interest in research activities, and is rather imposed by factors embedded in the training curriculum. This demonstrates that enforced participation does not induce sustainable interest in research beyond the residency training period. This also underscores the need for active interventions by all stakeholders in health research to implement measures to overcome the identified barriers to health research activity.

Despite the advantages conferred by the multicentre nature and high response rate of this study, the conclusions drawn from this study are limited by its conduct at a single time, inherent in its cross-sectional design, and its reliance on self-report with the associated likelihood of bias [3,6,12]. Furthermore, the extrapolation of findings is limited by the exploratory pilot design of the survey. Nevertheless, we believe that the present results should stimulate future nationwide longitudinal studies to confirm and extend these findings. In addition, other methods could be considered for data collection.

## Conclusions

The majority of trainee residents in Enugu, south-eastern Nigeria, were not currently participating in health research. Senior residents, postgraduate research training, previous participation in research and previous research publication were significantly associated with current participation in health research. The usefulness of health research in patient care, job promotion and understanding of disease are significant incentives for health research, while a lack of dedicated research time, poor funding and limited mentorship were perceived barriers to health research. Pre-residency research exposure, regular research training, provision of dedicated research time, adequate research funding and adequate rewards for mentorship are recommended to encourage participation in health research. Longitudinal studies are needed to establish the temporal trends in residents’ participation in research.

## Competing interests

The authors declare that they have no competing interests.

## Authors’ contributions

BIE conceived the study, designed the questionnaire, participated in data collection, analysis and interpretation, and wrote the manuscript. CN, JA, ENA, AM and FO participated in data collection, analysis and interpretation, made substantial contribution to the intellectual content of the manuscript, and approved the final version of the manuscript for submission. All authors read and approved the final manuscript.

## Pre-publication history

The pre-publication history for this paper can be accessed here:

http://www.biomedcentral.com/1472-6920/12/40/prepub
